# Dissociation of somatic growth, time of sexual maturity, and life expectancy by overexpression of an RGD‐deficient IGFBP‐2 variant in female transgenic mice

**DOI:** 10.1111/acel.12413

**Published:** 2015-10-28

**Authors:** Andreas Hoeflich, Anja Reyer, Daniela Ohde, Nancy Schindler, Julia Brenmoehl, Marion Spitschak, Martina Langhammer, Armin Tuchscherer, Elisa Wirthgen, Ingrid Renner‐Müller, Rüdiger Wanke, Friedrich Metzger, Maximilian Bielohuby, Eckhard Wolf

**Affiliations:** ^1^ Institute for Genome BiologyLeibniz Institute for Farm Animal Biology (FBN)18196DummerstorfGermany; ^2^ Institute for Genetics and BiometryLeibniz Institute for Farm Animal Biology (FBN)18196DummerstorfGermany; ^3^ Ligandis GbR18276Gülzow‐PrüzenGermany; ^4^ Institute of Molecular Animal Breeding and BiotechnologyGene CenterLMU Munich81377MunichGermany; ^5^ Institute of Veterinary PathologyLMU Munich80539MunichGermany; ^6^ F. Hoffmann‐La Roche Ltd.pREDPharma Research & Early DevelopmentDTA Neuroscience4070BaselSwitzerland; ^7^ Endocrine Research UnitMedizinische Klinik und Poliklinik IVKlinikum der Universität80336MunichGermany; ^8^ German Center for Diabetes Research (DZD)85764NeuherbergGermany

**Keywords:** growth, aging, reproductive development, cell signaling, IGFBP‐2, AKT

## Abstract

Impaired growth is often associated with an extension of lifespan. However, the negative correlation between somatic growth and life expectancy is only true within, but not between, species. This can be observed because smaller species have, as a rule, a shorter lifespan than larger species. In insects and worms, reduced reproductive development and increased fat storage are associated with prolonged lifespan. However, in mammals the relationship between the dynamics of reproductive development, fat metabolism, growth rate, and lifespan are less clear. To address this point, female transgenic mice that were overexpressing similar levels of either intact (D‐mice) or mutant insulin‐like growth factor‐binding protein‐2 (IGFBP‐2) lacking the Arg‐Gly‐Asp (RGD) motif (E‐ mice) were investigated. Both lines of transgenic mice exhibited a similar degree of growth impairment (−9% and −10%) in comparison with wild‐type controls (C‐mice). While in D‐mice, sexual maturation was found to be delayed and life expectancy was significantly increased in comparison with C‐mice, these parameters were unaltered in E‐mice in spite of their reduced growth rate. These observations indicate that the RGD‐domain has a major influence on the pleiotropic effects of IGFBP‐2 and suggest that somatic growth and time of sexual maturity or somatic growth and life expectancy are less closely related than thought previously.

## Introduction

In laboratory animals, growth inhibition by targeted mutation of the growth hormone or insulin‐like growth factor receptors (GHR, IGF1R) is known to significantly increase lifespan (Coschigano *et al*., 2003; Holzenberger *et al*., 2003), suggesting that higher growth activities may be at the expense of lifespan; this raises the question of whether smaller body dimensions might result in a healthier condition (Bartke, 2012). In addition, in worms and insects, long‐lived mutants with impaired insulin/IGF‐signaling frequently represent dwarf individuals (Kenyon, 2005). Within a species, for example, in dog breeds, size is negatively correlated with life expectancy, whereas between species, this correlation is, as a rule, positive (Kraus *et al*., 2013). However, phenotypic characteristics in long‐lived and dwarf mutant mice frequently have revealed reproductive deficits as well (Brown‐Borg, 2009), and experimental ablation of the reproductive system in nematodes has been found to increase both growth and lifespan (Patel *et al*., 2002). Consequently, there is an intriguing connection between somatic growth, reproductive development and lifespan. Currently, the strength of correlation between the different pleiotropic functions is unclear.

From the negative correlation between growth and life expectancy, it may be assumed that blocking the trophic effects of GH or IGF1 could be beneficial for healthy aging and an increased lifespan (Gallagher & LeRoith, 2011). In fact, female *Ghr* knockout mice in a C57BL/6 background have been demonstrated to have a 21% increased lifespan in comparison with wild‐type mice (Coschigano *et al*., 2003). In a different genetic background, *Ghr* ^−/−^ mice were able to reach an age of almost 5 years, which represents a quantum leap of lifespan extension and is unmatched by other long‐lived mouse models (Junnila *et al*., 2013). Owing to mutations of the GHR in Laron dwarfs and corresponding mouse mutants being associated with a drastic reduction of growth, it was investigated whether moderate growth impairment, for example, caused by overabundance of IGF1 inhibitory binding proteins (Hoeflich *et al*., 2004), may still have significant consequences for life expectancy and sexual development. Consequently, two different IGFBP‐2 transgenic mouse models (Hoeflich *et al*., 1999, 2002) overexpressing either wild‐type mouse IGFBP‐2 (D‐mice) or mutated IGFBP‐2 lacking the RGD‐motif required for integrin binding (Pereira *et al*., 2004) and carrying an RGE‐sequence instead (E‐mice) were investigated. Circulating IGF1 levels and IGFBP‐2 transgene expression levels in different tissues and compartments were similar in both transgenic lines, and this was due to the fact that identical expression vectors were used for generation of both mouse models (Hoeflich *et al*., 2002). The oncogenic potential of IGFBP‐2 for glioma progression is mediated by the RGD‐motif, integrin β1, and integrin‐linked kinase and results in the activation of the NF‐κB network (Holmes *et al*., 2012). Also, in breast cancer, IGFBP‐2 exerts malignant potential, and again, this depends on the RGD‐motif but involves regulation of estrogen receptors as demonstrated by the Perks laboratory (Foulstone *et al*., 2013). In addition, the RGD‐domain has been discussed in a context with metabolic functions of IGFBP‐2 (Wheatcroft & Kearney, 2009). In fact, by direct comparison of D‐ and E‐mice, we were able to demonstrate that the RGD‐motif present in IGFBP‐2 is involved in the regulation of GLUT4 trafficking and glucose clearance after oral glucose administration (Reyer *et al*., 2015). The RGD‐motif is distinct from the two IGF‐binding sites and both variants of IGFBP‐2 bind IGF2 with high affinity as demonstrated by Western ligand blotting (Hoeflich *et al*., 1999, 2002). Here, our experimental system was used for testing other pleiotropic functions of wild‐type vs. mutant IGFBP‐2 overabundance on somatic growth, time of sexual maturity, and lifespan. In particular, the hypotheses tested included i) moderate growth inhibition by IGFBP‐2 can prolong life expectancy significantly; and ii) the RGD‐sequence of IGFBP‐2 is a determinant of its pleiotropic effects. The results are discussed with respect to the original *antagonistic pleiotropy* concept (Williams, 1957) as well as to the more recent *disposable soma theory* of aging (Kirkwood & Austad, 2000).

## Results

**Figure 1:**
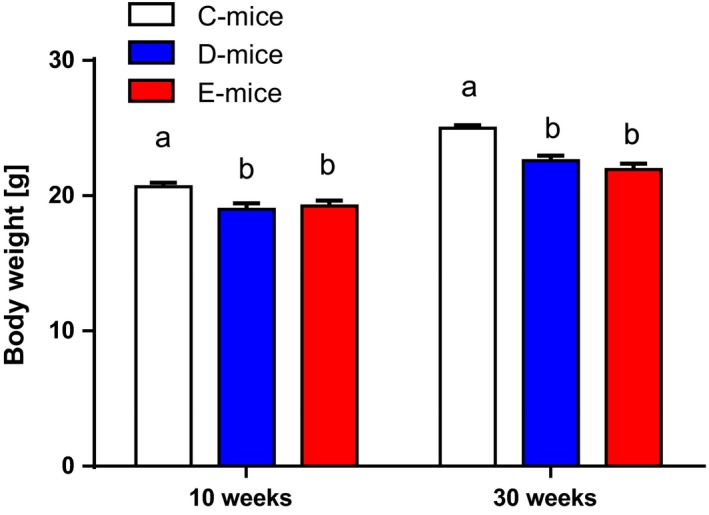
Body weights of female mice expressing wild‐type (D‐mice) or mutated IGFBP‐2 (E‐mice), and of nontransgenic controls (C‐mice) at two different ages (10 and 30 weeks). Both intact and mutated IGFBP‐2 exerted negative effects on somatic growth in 10‐ and 30‐week‐old female mice. Different superscripts indicate significant differences (*P* < 0.05). Data are presented as LSM ± SE (*n* > 14).

**Figure 2:**
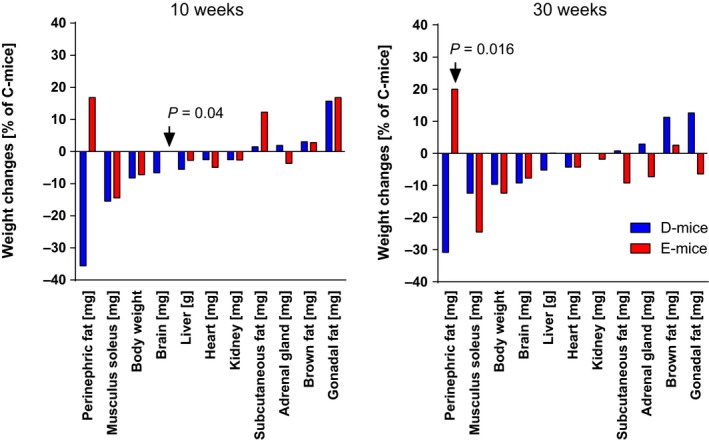
Organ weights of female mice expressing wild‐type (D‐mice) or mutated IGFBP‐2 (E‐mice), and of nontransgenic controls (C‐mice) at two different ages (10 and 30 weeks). Weight changes in transgenic mice are expressed as a percent increase or decrease in comparison with C‐mice, respectively (*n* > 14). Absolute values are summarized by Tables S1–S4.

**Figure 3:**
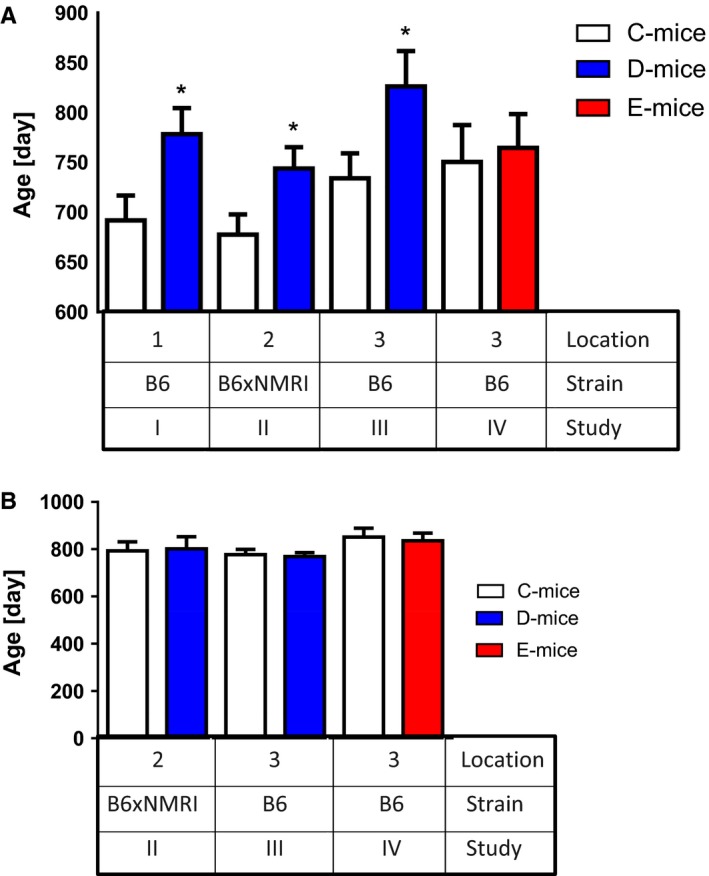
Life expectancy of female (A) and male (B) transgenic mice overexpressing wild‐type IGFBP‐2 (D‐mice) or IGFBP‐2 lacking the RGD‐sequence (E‐mice) compared to wild‐type controls (C). In three different locations and in the presence of different genetic backgrounds after an age of 490 days, female D‐mice were characterized by increased life expectancy compared with wild‐type littermates (*n*_c_: study I: 22, study II: 40, study III: 17; *n*_tg_: study I: 20, study II: 35, study III: 9). In study IV, female E‐mice (*n* = 19) were characterized by a similar long‐term survival in comparison with nontransgenic littermates (*n* = 16). Males: *n*_c_: study II: 17, study III: 20, study IV: 18; *n*_tg_: study II: 15, study III: 25, study IV: 16; data are presented as LSM ± SE; *P < 0.05.

**Figure 4:**
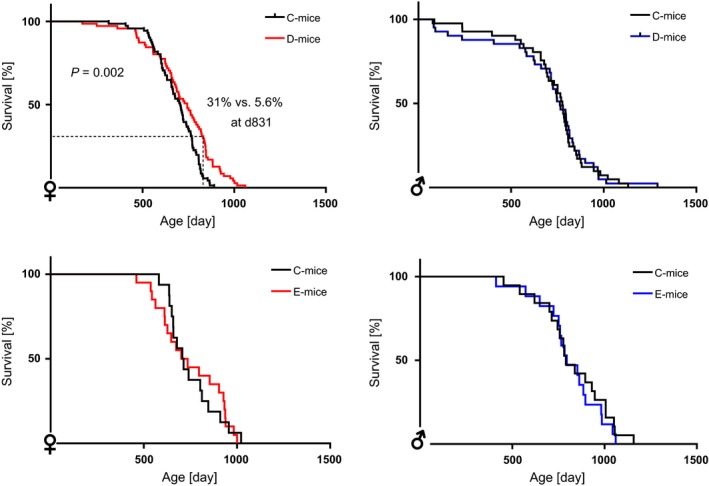
Progressive survival in female transgenic mice overexpressing wild‐type IGFBP‐2 (D‐mice) and in mice overexpressing IGFBP‐2 lacking the RGD‐sequence (E‐mice). Progressive survival was monitored in male and female D‐ and E‐mice versus controls from all locations included in the present study. Female D‐mice had increased survival (*P* = 0.002) in comparison with female C‐ mice: *n* = 71, respectively; male D‐ and C‐ mice: *n* = 41; female E‐mice and C‐mice: *n* = 16/19; male E‐ and C‐mice: *n* = 19/16 (B6: C57BL/6).

**Figure 5:**
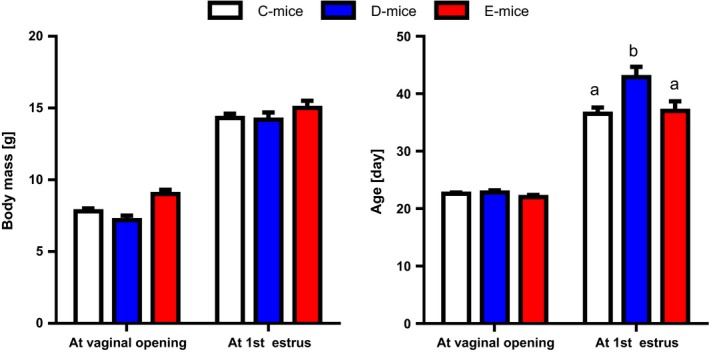
Reproductive development in female D‐ and E‐mice in comparison with controls. Plugs and vaginal smears were inspected at daily intervals and body weights were recorded. Data are presented as LSM ± SE (C‐mice: *n* = 45; D‐mice *n* = 17; E‐mice: *n* = 17). Different superscripts indicate significant differences with *P* < 0.05.

**Figure 6:**
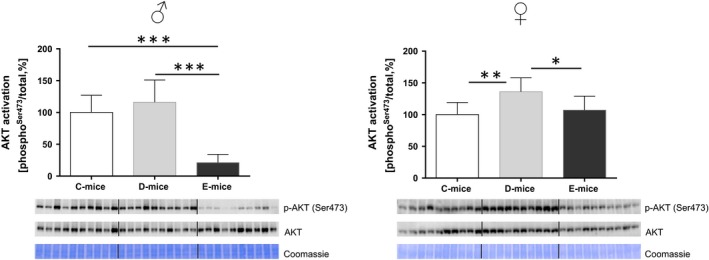
Specific activation of AKT in brain lysates from female D‐mice in comparison with E‐mice and controls at an age of 10 weeks. Data are presented as % of control ± SE *n* = 10; *P < 0.05; **P < 0.01; ***P < 0.001.

The inhibitory effects of wild‐type and RGD‐deficient IGFBP‐2 on somatic growth observed at 10 weeks of age were also persistent in 30‐week‐old female mice (Fig. 1). In both age groups, the weight reductions of D‐mice and E‐mice in comparison with C‐mice ranged between 8% and 13%. On a tissue level (Fig. 2), overexpression of wild‐type IGFBP‐2 in female D‐mice resulted in stronger weight reductions in skeletal muscles than in the liver, heart, or brain, which is in accordance with previous results (Hoeflich *et al*., 1999). At 10 weeks of age, the effects of RGD‐deficient IGFBP‐2 in E‐mice were comparable to those in D‐mice for body weight and weights of isolated muscle, liver, heart, perinephric, brown fat, and gonadal fat. At 10 weeks of age, the negative effect of IGFBP‐2 on the brain weight observed in D‐mice was not reproduced in E‐mice. Perinephric fat mass was particularly sensitive to the inhibitory effects of wild‐type IGFBP‐2 overexpression. At an age of 30 weeks, the effects of wild‐type and mutant IGFBP‐2 overexpression were significantly different. The absolute weight data and pairwise comparisons for both age groups are provided as Tables S1–S4. As identified by three independent studies in three different mouse facilities including 78 transgenic mice and 63 controls (Fig. 3A), overexpression of wild‐type IGFBP‐2 resulted in a significant increase of long‐term survival in female D‐mice (*P* < 0.05). The beneficial effects of elevated IGFBP‐2 expression for maximal life length were observed in C57BL/6 (study I and III) and in mice of a mixed genetic background (50% C57BL/6, 50% NMRI; study II). Consequently, this increase is not restricted to one defined inbred strain but is also present in a more complex genetic background. While growth restrictions were also present in male mice, ranging between 9% and 11% (Tables S2 and S4), in contrast to their female counterparts, male D‐mice and E‐mice had no increase in lifespan compared to sex‐matched C‐mice (Fig. 3B). In addition, progressive survival was higher (*P* = 0.002) in female but not in male D‐mice (Fig. 4). While at an age of 831 days, 31% of all female D‐mice were still alive, less than 6% of nontransgenic littermates had survived. However, the effects of IGFBP‐2 on lifespan were dependent on the presence of the RGD‐sequence in IGFBP‐2 because in E‐mice, no life‐prolonging effect was observed (study IV; lifespan E‐mice: 765 ± 34 days, *n* = 19; lifespan C‐mice: 750 ± 37 days, *n* = 33). It was next considered if reproductive development was altered in D‐ or E‐mice. In fact, female D‐ but not E‐mice were characterized by significantly delayed sexual maturity (Fig. 5). While in control mice, age at first estrus was 36.1 ± 1.1 days, D‐mice reached an age of 42.9 ± 1.8 days before they came into their first estrus. By contrast, a delay of reproductive development was not found in E‐mice (E‐mice; 37.0 ± 1.7 days). Age at vaginal opening was unaffected by the genotype. At an age of 10 weeks phosphorylation of Ser473 present in AKT was specifically increased in female brain lysates (Fig. 6) but not in liver, fat, or muscle tissues (data not shown) from D‐mice compared to E‐mice and controls.

## Discussion

In this study, the pleiotropic effects of intact and RGD‐deficient IGFBP‐2 were investigated in transgenic mice. Whether the RGD‐motif present in IGFBP‐2 is relevant for growth, reproductive development and lifespan were considered. In particular, the strength of correlation between impaired somatic growth and improved long‐term survival observed was assessed in a number of mouse models characterized by growth inhibition (Junnila *et al*., 2013).

Mild growth restrictions were found in mice overexpressing wild‐type or mutant IGFBP‐2 lacking the RGD‐motif. These findings are consistent with previous results (Hoeflich *et al*., 1999, 2002) and support the hypothesis that the RGD‐motif is not required for the negative effects of IGFBP‐2 on somatic growth. We have shown previously that IGFBP‐2 also is a potent inhibitor of bone mass and bone lengths (Eckstein *et al*., 2002) thus somatic growth inhibition is found both for body mass and skeletal parameters. On a tissue level, it was found that the RGD‐sequence is of relevance in the brain and in perinephric fat, which can be observed because age‐specific effects in D‐ versus E‐mice have been observed in these tissues. The weight effects of IGFBP‐2 overexpression in other peripheral fat depots were not very well reproduced, particularly in 30‐week E‐mice. Consequently, it is necessary to assume that there are age‐ and tissue‐dependent effects of the RGD‐domain present in IGFBP‐2 for the control of fat accretion. At younger ages, overexpression of mouse IGFBP‐2 increased gonadal fat mass (Rehfeldt *et al*., 2010), whereas overexpression of human IGFBP‐2 in mice blocked the development of age‐related obesity later in life (Wheatcroft *et al*., 2007). The latter cannot be observed in the model included in this study; instead, these results indicate differential effects of mouse IGFBP‐2 in perinephric and other fat tissues. Currently, a role of two distinct heparin‐binding domains present in IGFBP‐2 has been suggested for adipocyte differentiation (Xi *et al*., 2013), and the RGD‐motif has not been considered as an effector of fat metabolism.

In general, the female C‐mice investigated in this study had a shorter lifespan when compared with male C‐mice, which is in line with previous results in C57BL/6 mice (Turturro *et al*., 1999). Growth inhibition in females but not in males correlated with improved long‐term survival in D‐mice. A sexually dimorphic effect on lifespan has also been described in heterozygous *Igf1r* knockout mice (Holzenberger *et al*., 2003) and fits with the concept that reduced IGF1 signaling affects lifespan, particularly in female mice, whereas impaired GH signaling increases lifespan in both sexes (Bartke, 2011; Junnila *et al*., 2013). In this study, *antagonistic pleiotropy* (Bartke, 2011), which describes beneficial and adverse effects of a hormone during ontogeny, was only observed in female D‐mice. Surprisingly, growth inhibition in female E‐mice was not associated with an increase in lifespan, as noted in female D‐mice. Consequently, loss of the RGD‐motif loosened the negative correlation between growth and lifespan.

Between species, early reproductive development is associated with a shorter lifespan (Harvey & Zammuto, 1985). Consequently, this study considered if reproductive development was altered in D‐ or E‐mice. Female D‐ but not E‐mice required significantly more time than C‐mice for reproductive development, which was measured as the age at first estrus. Preliminary data further indicate significant reductions of ovary weights in D‐ but not in E‐mice at an age of 10 weeks (data not shown). Therefore, the findings in this study support the idea of a link between lifespan and reproductive development. Ablation of the reproductive system in worms or insects has been demonstrated to extend lifespan (Rose & Charlesworth, 1980; Hsin & Kenyon, 1999) supporting the *disposable soma theory* of aging, which reflects the functional interrelationships between the reproductive system and other cells or tissues (Kirkwood & Austad, 2000). One central assumption of that theory is that energetic input in nonreproductive tissues has to be at the expense of cells required for reproduction and vice versa owing to a certain limitation of resources. While the *disposable soma theory* may be in line with higher lifespan in growth‐restricted animals, it does not explain the lack of lifespan effects in growth‐restricted E‐mice observed in the present study. By contrast, these findings are not contradictory to the more general concept of *antagonistic pleiotropy* (Williams, 1957) according to which, specific factors may have beneficial and detrimental effects depending on the period of time. However, delayed reproductive development may also correlate with somatic overgrowth in mice overexpressing LIN28B (Zhu *et al*., 2010), pointing to the fact that the connections between growth and reproductive development may not be unidirectional. Both somatic growth and reproductive development do represent early events in individual ontogeny. However, both events have differential dynamics in D‐, E‐, and C‐mice. This implicates, at least to some extent, that an uncoupling of pleiotropic IGFBP‐2 effects is possible in the current experimental system. The concept of *antagonistic pleiotropy* has been discussed extensively in the context of the GH/IGF‐system before (Bartke, 2011).

In addition, it is also necessary to consider particular current concepts of an interrelation between lifespan, reproduction, and fat metabolism (Hansen *et al*., 2013). It is well established that estradiol signaling is centrally connected with reproduction and energy metabolism (Sinchak & Wagner, 2012). In that context, it is of particular interest that overexpression of the IGFBP‐2 variant inversed the effects of wild‐type IGFBP‐2 in peripheral fat pads in mice. Consequently, the system in this study supports the idea of a connection between fat metabolism, reproductive development, and life expectancy. Foulstone and coworkers found that IGFBP‐2 controls estrogen receptor alpha expression in breast cancer cells (Foulstone *et al*., 2013). Thus, the dynamics of reproductive development may represent a determinant of lifespan, as originally observed by interspecies comparisons and after correction for body weight as a confounding factor (Harvey & Zammuto, 1985). In addition, in mutant laboratory mouse strains, delayed maturation was associated with higher lifespan (Brown‐Borg, 2009). However, all models had reduced body dimensions in common. Here, it was demonstrated that lifelong reduction of somatic growth is not sufficient to increase the length of life. Instead, these findings support the concepts that integrate reproductive development, energy metabolism, and lifespan, which have already been demonstrated in worms and flies (Gerisch *et al*., 2001; Flatt *et al*., 2008). Imaginal morphogenesis protein‐late 2 (IMP‐L2), a protein homologous to MAC25 (IGFBP7) that binds to insulin in Drosophila, has also been demonstrated to increase lifespan (Honegger *et al*., 2008; Alic *et al*., 2011), and it was discussed that IGFBPs may play a role in mammalian aging. Furthermore, IMP‐L2 is increased in germline‐less flies (Flatt *et al*., 2008), which may be connected with improved longevity and thus suggests that insulin‐ or the IGF‐binding protein may have conserved functions during evolution.

It is of particular interest that genetic linkage of female reproductive development and lifespan has been suggested to be mediated by IGF1 in inbred mouse lines (Yuan *et al*., 2012). In this work, evidence is provided for the coupling of pleiotropic functions of IGFBP‐2 being mediated by the RGD‐motif, which is primarily considered to be IGF‐independent (Wheatcroft & Kearney, 2009). However, coupling of reproductive development and lifespan has been clearly demonstrated in *Caenorhabditis elegans* (Dillin *et al*., 2002). Thus, while this study demonstrates uncoupling of somatic growth and the control of lifespan, it does not exclude the possibility that, in other models, functional coupling of both parameters may occur. In addition, we cannot exclude that in a condition of stronger body weight reduction functional coupling of somatic growth and lifespan may be observed. Concerning the control of circulating versus local IGF‐activity, we may speculate that local effects of IGF1 may be more important than endocrine effects at least in our experimental system, as both IGFBP‐2 variants bind the IGFs with high affinity, whereas tissue‐specific affects are different due to the presence or absence of integrin binding via the RGD‐motif. Our results further support a functional relevance of the RGD‐motif for activation of AKT in female brains.

In summary, it was demonstrated that growth inhibition per se does not increase the lifespan. In terms of pleiotropy, this study provides evidence that isolated functions of IGFBP‐2 rely on the presence of the RGD‐motif present in IGFBP‐2. These functions include reproductive development, accretion of fat mass, and long‐term survival. While these results are not superimposable with the *disposable soma theory* of aging, the concept of *antagonistic pleiotropy* may integrate with the results. Accordingly, reproductive development and energy metabolism, but less stringent somatic growth, impact on the control of long‐term survival.

## Materials and methods

### Animal experiments

All animal experiments were performed in accordance with the German Animal Welfare Act with permission from the respective animal welfare authorities. Mice were kept in standard cages and had access to pelleted food (studies I and II: V1534, Ssniff, Soest, Germany; studies III and IV: Altromin 1314, Altromin, Lage, Germany) and water *ad libitum*. For studying long‐term survival, mice older than 490 days were used. In study I, male and female hemizygous IGFBP‐2 transgenic mice and controls (C57BL/6) as described in (Hoeflich *et al*., 1999) were kept at the Gene Center, LMU Munich. In study II, IGFBP‐2 transgenic mice and controls were used from a mixed genetic background (C57BL/6 × NMRI) (Hoeflich *et al*., 2001) and were maintained in the mouse facility of the Institute of Veterinary Pathology (LMU Munich). Studies III and IV were performed under semi‐barrier conditions in the mouse facility of the Leibniz Institute of Farm Animal Biology (FBN) in Dummerstorf, Germany. In both studies, transgenic C57BL/6 mice overexpressing either wild‐type IGFBP‐2 containing the intact RGD‐sequence (D‐mice; study III) or mutant IGFBP‐2 (E‐mice; study IV) lacking the RGD‐sequence motif (Hoeflich *et al*., 2002) were used. If senescent mice were suffering from severe illness or had any other signs of distress, they were euthanized. For the determination of sexual maturity, vaginal smears were stained with hematoxylin and eosin before being visualized under a microscope at daily intervals. The initial presence of cornified vaginal epithelial cells lacking nuclear staining was considered as the day of first estrus (Allen, 1922). At an age of 10 and 30 weeks, mice were euthanized by cervical dislocation, organs were dissected, and their weights were recorded. Analysis of signal transduction was performed as recently described (Reyer *et al*., 2015).

### Statistical analysis

The data analysis was performed using SAS software, version 9.4 for Windows (Copyright, SAS Institute Inc., Cary, NC, USA). Descriptive statistics and tests for normality were carried out using the UNIVARIATE procedure of Base SAS software. Data that could be considered as approximately normally distributed were analyzed by ANOVA with the MIXED procedure of SAS/STAT software. The ANOVA model for the body and organ weight data contained the fixed factors genotype (levels: C, D, and E), age (levels: 10, 30 weeks) and sex (levels: male, female), and all interactions between the fixed factors.

The lifespan data were analyzed by one‐way ANOVA using models with the factors transgene (levels: C, D, or levels: C, E) for each genotype, line, study, and sex (c.f. studies I–IV). In these analyses, only animals with a minimum age of 490 days were considered. In addition, the survivor functions were estimated and compared with the LIFETEST procedure of SAS/STAT software and the Mantel‐Cox test (GraphPad Prism software, GraphPad, la Jolla, CA, USA). A one‐way model with the factor genotype (levels: C, D, E) was applied for the maturation data. Least‐square means (LSM) and their standard errors (SE) were computed for each fixed effect in the ANOVA models, and all pairwise differences of LSM were tested for significance using the Tukey–Kramer procedure. Effects and differences were considered significant if *P* < 0.05.

## Funding

This work was supported by a grant from the Deutsche Forschungsgemeinschaft (DFG HO 2003/6‐1). DO was supported by a grant from the H. Wilhelm Schaumann Stiftung.

## Author contributions

AH, AR, IRM, RW, and EWo designed the animal models and the study. All authors performed experiments, analyzed data, and interpreted the results. AH wrote the manuscript including comments from all authors.

## Conflict of interest

The authors declare no competing financial interests. FM and EWi are employed by F. Hoffmann‐La Roche Ltd. and Ligandis Biomarker Service, respectively; however, this does not influence the interpretation of the results. The authors declare that this work has not been submitted elsewhere. All authors have declared their consent with the content of the manuscript.

## Supporting information


**Table S1** Body and organ weights in female mice at an age of 10 weeks.
**Table S2** Body and organ weights in male mice at an age of 10 weeks.
**Table S3** Body and organ weights in female mice at an age of 30 weeks.
**Table S4** Body and organ weights in male mice at an age of 30 weeks.
