# Supplementary material for: Dissociation of somatic growth, time of sexual maturity, and life expectancy by overexpression of an RGD‐deficient IGFBP‐2 variant in female transgenic mice
Source: Aging Cell. 2015 Oct 28;15(1):111–7. doi: 10.1111/acel.12413 (PMC4717279; doi:10.1111/acel.12413)
Supplement: Supplementary file 1 [file ACEL-15-111-s001.docx]

Supplemental table 1: Body and organ weights in female mice at an age of 10 weeks. Data are presented as least square means (LSM) and SE. Group comparisons have been performed between D- and C-mice (D/C), E- and C-mice (E/C) and D- and E-mice (D/E). Adjusted P-values are <0.05 (*). Subcutan.: subcutaneous; M.: musculus.

	C		D		E		comparison		
	LSM	SE	LSM	SE	LSM	SE	D/C	E/C	D/E
body weight [g]	20.7	0.3	19.0	0.4	19.2	0.4	*	0.052	
adrenal gland [mg]	7.21	0.28	7.35	0.41	6.94	0.38			
brown fat [mg]	63.5	3.6	65.5	5.3	65.3	4.8			
brain [mg]	420.5	4.7	392.9	6.9	420.4	6.3	*		*
heart [mg]	98.5	2.2	96.0	3.3	93.7	3.0			
kidney [mg]	244.9	4.8	238.7	7.1	238.5	6.5			
perinephric fat [mg]	41.0	7.3	26.4	10.8	47.9	9.9			
liver [g]	1.10	0.03	1.04	0.05	1.07	0.05			
Subcutan. fat [mg]	263.2	26.5	267.2	39.4	295.6	35.9			
M. soleus [mg]	209.6	6.0	177.4	8.8	179.5	8.1	*	*	
gonadal fat [mg]	20.7	0.3	19.0	0.4	19.2	0.4			

Supplemental table 2: Body and organ weights in in male mice at an age of 10 weeks. Data are presented as least square means (LSM) and SE. Groupwise comparisons have been performed between D- and C-mice (D/C). E- and C-mice (E/C) and D- and E-mice (D/E). Adjusted P-values are <0.05 (*) and <0.01 (**). Subcutan.: subcutaneous; M.: musculus.

	C		D		E		comparison		
	LSM	SE	LSM	SE	LSM	SE	D/C	E/C	D/E
body weight [g]	24.4	0.5	21.9	0.6	22.8	0.6	**		
adrenal gland [mg]	5.44	0.23	5.74	0.29	5.21	0.30			*
brown fat [mg]	75.9	5.5	77.8	6.7	79.4	7.4			
brain [mg]	426.0	5.0	389.1	6.3	423.7	6.7	**		**
heart [mg]	113.7	2.8	102.0	3.5	108.4	3.7			
kidney [mg]	323.2	8.5	290.6	10.8	304.6	11.3			
perinephric fat [mg]	40.6	19.3	39.6	23.4	40.8	26.0			
liver [g]	1.26	0.04	1.29	0.05	1.34	0.05			
suboutan. fat [mg]	332.6	44.5	313.8	56.6	320.6	59.5			
M. soleus [mg]	281.2	8.7	231.2	11.1	246.3	11.7	**		
gonadal fat [mg]	240.5	34.6	222.9	44.1	240.8	46.3			

Supplemental table 3: Body and organ weights in female mice at an age of 30 weeks. Data are presented as least square means (LSM) and SE. Group comparisons have been performed between D- and C-mice (D/C), E- and C mice (E/C) and D- and E-mice (D/E). Adjusted P-values are <0.05 (*), <0.01 (**) and <0.001 (***). Subcutan.: subcutaneous; M.: musculus.

	C		D		E		comparison		
	LSM	SE	LSM	SE	LSM	SE	D/C	E/C	D/E
body weight [g]	25.0	0.2	22.6	0.4	21.9	0.4	***	***	
adrenal gland [mg]	7.93	0.23	8.16	0.36	7.35	0.48			
brown fat [mg]	65.8	3.1	74.1	4.6	61.6	6.2			
brain [mg]	433.4	3.8	414.7	6.0	414.9	8.1			
heart [mg]	115.6	1.8	109.6	2.8	115.7	3.8			
kidney [mg]	287.3	3.9	287.2	6.2	282.1	8.3			
perinephric fat [mg]	101.6	6.4	70.3	9.4	121.9	12.7			*
liver [g]	1.19	0.03	1.20	0.04	1.08	0.06			
subcutan. fat [mg]	555.8	24.7	504.5	46.0	513.1	68.2			
M. soleus [mg]	283.3	4.9	248.2	7.7	213.8	10.3	**	***	
gonadal fat [mg]	291.2	15.0	323.7	23.5	298.6	31.6			

Supplemental table 4: Body and organ weights in male mice at an age of 30 weeks. Data are presented as least square means (LSM) and SE. Groupwise comparisons have been performed between D- and C-mice (D/C). E- and C-mice (E/C) and D- and E-mice (D/E). Adjusted P-values are <0.1 (#), <0.01 (**) and <0.001 (***). Subcutan.: subcutaneous; M.: musculus.

	C		D		E		comparison		
	LSM	SE	LSM	SE	LSM	SE	D/C	E/C	D/E
body weight [g]	31.4	0.3	28.0	0.4	28.8	0.4	***	***	
adrenal gland [mg]	4.79	0.15	4.44	0.20	5.37	0.22			
brown fat [mg]	110.9	4.4	111.6	5.4	103.2	6.1			
brain [mg]	427.7	3.2	400.5	4.4	417.9	4.9	***		#
heart [mg]	141.5	1.8	137.6	2.4	140.5	2.7			
kidney [mg]	405.5	5.5	400.5	7.5	406.2	8.4			
perinephric fat [mg]	235.1	15.3	193.1	19.0	234.9	21.5			
liver [g]	1.56	0.03	1.54	0.04	1.50	0.04			
subcutan. fat [mg]	753.0	32.4	685.7	47.4	669.4	46.6			
M. soleus [mg]	330.1	5.7	266.5	7.7	286.1	8.6	***	**	
gonadal fat [mg]	561.3	22.6	537.3	30.4	509.8	34.1			
